# A Prospective Observational Study on Maternal and Foetal Outcomes Among Primigravid Women With High Body Mass Index in a Tertiary Care Hospital in West Bengal

**DOI:** 10.7759/cureus.102141

**Published:** 2026-01-23

**Authors:** Kamal K Dash, Jayeeta Burman, Biplab Bala, Sembagamuthu Sembiah

**Affiliations:** 1 Gynaecology and Obstetrics, Sarat Chandra Chattopadhyay Government Medical College and Hospital, Uluberia, IND; 2 Community Medicine, Sarat Chandra Chattopadhyay Government Medical College and Hospital, Uluberia, IND; 3 Community Medicine and Family Medicine, All India Institute of Medical Sciences, Kalyani, Kalyani, IND

**Keywords:** hypertensive disorders of pregnancy, maternal obesity, neonatal morbidity, pregnancy outcomes, primigravid

## Abstract

Context

Overweight and obesity among Indian women of reproductive age are increasing, posing important risks for pregnancy, particularly in primigravid women. Evidence focused solely on primigravid women remains limited.

Aim

This study aims to assess maternal and foetal outcomes among primigravid women with high body mass index (BMI) attending a tertiary care hospital in Kolkata.

Settings and design

This is a prospective observational study conducted over one year in a tertiary care obstetric unit.

Methods and materials

A total of 144 primigravid women enrolled at ≤12 weeks of gestation were categorised into normal BMI (18.5-24.9 kg/m²) and high BMI (≥25 kg/m²). Maternal outcomes included hypertensive disorders of pregnancy (HDP), gestational diabetes mellitus (GDM), labour and delivery complications. Foetal outcomes assessed were preterm birth, neonatal morbidity, and NICU admission. Logistic regression was used to estimate adjusted odds ratios (AOR).

Results

A high BMI was present in 40.3% of participants. High BMI was significantly associated with HDP and GDM. Labour complications and delivery complications were also more frequent. Infants of high-BMI mothers showed higher odds of being born preterm and of NICU admission.

Conclusions

High early-pregnancy BMI is an important predictor of adverse maternal and neonatal outcomes in primigravid women. Early BMI screening, focused antenatal surveillance, and preconception counselling are essential for risk reduction.

## Introduction

Obesity in women of reproductive age has emerged as a major clinical concern with significant implications for maternal and perinatal health. The global rise in overweight and obesity over recent decades reflects profound lifestyle and nutritional transitions, and increasing numbers of women are now entering pregnancy with elevated body mass index (BMI). According to the World Health Organization (WHO), more than 650 million adults worldwide are obese, with a substantial proportion being women in their reproductive years [1]. In India, findings from the National Family Health Survey-5 (NFHS-5, 2019-2021) indicate that nearly one-fourth of women aged 15-49 years are overweight or obese [2], highlighting a growing burden that intersects directly with maternal health services. A 2024 systematic review reported obesity prevalence ranging from 40% to 46% among pregnant women in certain populations worldwide [3]. According to NFHS-4 data, the national prevalence of obesity among pregnant and postpartum women was around 12-13%, with several districts reporting rates above 40%, underscoring significant regional variation and a growing public health burden [4].

Excess adiposity is known to disrupt normal physiological adaptation to pregnancy. Obesity is associated with chronic low-grade inflammation, insulin resistance, endothelial dysfunction, and altered placental development, which collectively increase susceptibility to metabolic and hypertensive complications of pregnancy [5,6]. Prior studies have also shown that high BMI adversely affects labour progress and delivery outcomes, including a higher likelihood of prolonged labour, induction failure, and operative delivery [7,8]. In addition, the intrauterine metabolic environment of obese mothers may influence foetal growth patterns and immediate neonatal well-being, contributing to increased risks of respiratory distress, neonatal hypoglycaemia, and need for intensive care [3,9].

Although evidence describing the impact of maternal obesity is expanding globally, data focusing specifically on primigravid women remain limited, particularly in the Indian context. Primigravidae represent a physiologically unique group, as they undergo pregnancy-related metabolic, vascular, and anatomical changes for the first time. Their risk profile may differ from that of multigravid women, and the influence of an elevated BMI may be more pronounced [10]. Understanding these associations in primigravidae is essential for informing targeted antenatal interventions and strengthening maternal care pathways.

In view of the rising prevalence of obesity among young women and the need to better characterise its impact on first pregnancies, the present study was undertaken to assess maternal and foetal outcomes among primigravid women with high BMI attending a tertiary care hospital in Kolkata.

## Materials and methods

Study design and setting

This was a prospective observational study conducted in the Department of Obstetrics and Gynaecology at a tertiary care hospital in Kolkata, West Bengal. The study period extended from November 2022 to November 2023. The hospital is a major tertiary referral centre serving a predominantly urban population and a high proportion of high-risk obstetric cases. Prior to commencement, approval was obtained from the Institutional Ethics Committee.

Study population

A total of 144 primigravid women were enrolled in the study. The study used convenience sampling, and all eligible primigravid women registering at ≤12 weeks of gestation during the study period were consecutively enrolled until the end of the study, yielding a final sample size of 144 participants. Participants were recruited during their first antenatal visit, provided they registered at ≤12 weeks of gestation. Early recruitment ensured that the booking BMI closely approximated the pre-pregnancy BMI, as recommended by the WHO. Women who were primigravida with singleton pregnancy, gestational age ≤12 weeks at booking, and who provided informed consent were included. Women with a BMI less than 18.5 kg/m², pre-existing diabetes mellitus, chronic hypertension, thyroid disorders diagnosed before pregnancy, bronchial asthma or other major medical illnesses, as well as those with multifoetal gestation or foetal anomalies detected antenatally, were excluded from the study.

Study variables

The primary independent variable was maternal BMI measured at the booking visit (≤12 weeks), categorised as normal (18.5-24.9 kg/m²) or high (≥25 kg/m²).

The dependent variables were the individual maternal and foetal outcomes assessed during the antenatal, intrapartum, and postnatal periods, including gestational hypertension, preeclampsia, gestational diabetes mellitus (GDM), induction of labour, mode of delivery, postpartum haemorrhage, preterm birth, neonatal respiratory distress, Apgar score, hypoglycaemia, and NICU admission.

Definitions of outcome variables

Maternal Outcomes

Maternal outcomes included hypertensive disorders of pregnancy (HDP), encompassing gestational hypertension (BP ≥140/90 mmHg after 20 weeks without proteinuria) and preeclampsia (gestational hypertension with proteinuria ≥300 mg/24 hours or organ dysfunction); GDM, defined as two-hour plasma glucose ≥140 mg/dL after a 75 g glucose load; induction of labour, referring to pharmacological or mechanical initiation of uterine contractions; failed or prolonged labour, defined as failure to achieve active labour or progress despite adequate uterine contractions for the recommended duration, or total labour duration exceeding accepted physiological limits as per WHO partograph standards; mode of delivery, including normal vaginal delivery, operative vaginal delivery (forceps or vacuum), and caesarean section (elective or emergency); postpartum haemorrhage (PPH), defined as blood loss >500 mL after vaginal delivery or >1000 mL after caesarean section within the first 24 hours postpartum (primary PPH); and wound infection occurring within 10 days postpartum, requiring local or systemic treatment.

Foetal and Neonatal Outcomes

Foetal and neonatal outcomes included preterm birth, defined as delivery before 37 completed weeks; low birth weight (LBW), defined as birth weight <2.5 kg; macrosomia, defined as birth weight ≥4.0 kg; neonatal respiratory distress, defined as the requirement of supplemental oxygen or ventilatory support within 24 hours of birth; Apgar score <7 at five minutes, defined as a score less than seven at five minutes postpartum indicating neonatal compromise requiring additional resuscitative measures; NICU admission, defined as the requirement for admission to the neonatal intensive care unit due to respiratory, metabolic, or other clinical instability immediately after birth; and neonatal hypoglycaemia, defined as blood glucose concentration <40 mg/dL in the first 24 hours of life requiring feeding intervention or intravenous glucose therapy.

Data collection and follow-up

Data were collected using a pretested structured proforma. Baseline variables, including maternal age, education, socioeconomic status (modified Kuppuswamy scale), and anaemia status, were recorded at the booking visit (≤12 weeks of gestation). No additional study-specific visits were scheduled, and participants were followed prospectively during routine antenatal care. Antenatal outcomes such as gestational hypertension, preeclampsia, and GDM were assessed throughout pregnancy during scheduled antenatal visits. Intrapartum outcomes, including induction of labour, labour progression, and mode of delivery, were documented at the time of labour and delivery. Postpartum maternal outcomes were assessed during the hospital stay and up to 6 weeks postpartum, while foetal and neonatal outcomes were recorded at birth and during the neonatal period until discharge or NICU admission, as applicable.

Statistical analysis

Data analysis was performed using IBM SPSS Statistics for Windows, Version 27.0 (IBM Corp., Armonk, NY). Quantitative data were summarised using mean ± standard deviation. Categorical variables were expressed as proportions and compared using the chi-square test. Logistic regression was performed to calculate crude odds ratios (ORs) for maternal and foetal complications and adjusted odds ratios (AORs) controlling for maternal age, socioeconomic status, anaemia status, and gestational age at delivery. Variables included in the multivariable model were selected a priori based on biological plausibility and evidence from previous studies. Maternal age, socioeconomic status, anaemia status, and gestational age at delivery were included as potential confounders, as these factors are known to be associated with both maternal BMI and pregnancy outcomes. A p-value < 0.05 was considered statistically significant.

## Results

A total of 144 primigravid women were included in the study, of whom 86 (59.7%) had normal BMI and 58 (40.3%) had high BMI. Most participants (66.7%) were aged 25 years or older, and the age distribution did not differ significantly between BMI groups (p = 0.21). The majority of women were urban residents (81.9%), and educational status and socioeconomic class were comparable between groups. Anaemia was present in 25% of the study population, with no significant difference across BMI categories. As expected, the mean first-trimester BMI was significantly higher in the high-BMI group (29.3 ± 2.1 kg/m² vs. 22.0 ± 1.4 kg/m², p < 0.001) (Table 1).

**Table 1:** Baseline characteristics of the study population

Characteristic	Total (N = 144) (values in %)	Normal BMI (n = 86)	High BMI (n = 58)	p-value
Maternal age (years)				
<25 years	48 (33.3)	32 (37.2)	16 (27.6)	0.21
≥25 years	96 (66.7)	54 (62.8)	42 (72.4)	-
Residence				
Urban	118 (81.9)	72 (83.7)	46 (79.3)	0.53
Rural	26 (18.1)	14 (16.3)	12 (20.7)	-
Education				
Up to secondary	52 (36.1)	33 (38.4)	19 (32.8)	0.49
Higher secondary and above	92 (63.9)	53 (61.6)	39 (67.2)	-
Socioeconomic status				
Upper/upper-middle	64 (44.4)	42 (48.8)	22 (37.9)	0.19
Middle/lower-middle	80 (55.6)	44 (51.2)	36 (62.1)	-
Anaemia				
Present	36 (25.0)	20 (23.3)	16 (27.6)	0.56
Absent	108 (75.0)	66 (76.7)	42 (72.4)	-
Gestational age at delivery in weeks, (mean ± SD)	38.2 ± 1.8	38.6 ± 1.6	37.7 ± 2.0	0.42
BMI at booking (mean ± SD)	24.9 ± 2.6	22.0 ± 1.4	29.3 ± 2.1	<0.001

The prevalence of gestational hypertension (17.2% vs. 7.0%) and preeclampsia (12.1% vs. 3.5%) was significantly higher in the high-BMI group. Similarly, GDM occurred more frequently among women with high BMI. High BMI was also associated with a significantly greater need for induction of labour and a higher incidence of caesarean section (31.0% vs. 14.0%). Although postpartum haemorrhage and wound infection were more common among high-BMI women, these differences were not statistically significant (Table 2).

**Table 2:** Distribution of study participants according to maternal outcomes ^*^p value < 0.05; statistically significant.

Maternal outcomes	Total (N = 144)	Normal BMI (n = 86)	High BMI (n = 58)	p
Gestational hypertension	16 (11.1)	6 (7.0)	10 (17.2)	0.048*
Preeclampsia	10 (6.9)	3 (3.5)	7 (12.1)	0.041*
Gestational diabetes mellitus (GDM)	14 (9.7)	5 (5.8)	9 (15.5)	0.049*
Induction of labour	28 (19.4)	12 (14.0)	16 (27.6)	0.042*
Caesarean section	34 (23.6)	12 (14.0)	22 (31.0)	0.012*
Postpartum haemorrhage	4 (2.8)	1 (1.2)	3 (5.2)	0.18
Wound infection	5 (3.5)	2 (2.3)	3 (5.2)	0.40

Foetal and neonatal outcomes are shown in Table 3. Preterm birth was significantly more common among high-BMI women (22.4% vs. 10.5%). Other adverse outcomes - including LBW, respiratory distress, neonatal hypoglycaemia, and low Apgar score - were more frequent in the high-BMI group, although not statistically significant. NICU admission was significantly higher in neonates born to high-BMI mothers (15.5% vs. 5.8%).

**Table 3:** Distribution of study participants according to foetal/neonatal outcomes ^*^p value < 0.05; statistically significant.

Foetal/neonatal outcomes	Total (N = 144)	Normal BMI (n = 86)	High BMI (n = 58)	p
Preterm birth (<37 weeks)	24 (16.7)	9 (10.5)	13 (22.4)	0.048*
Low birth weight (<2.5 kg)	8 (5.6)	5 (5.8)	3 (5.2)	0.91
Respiratory distress	8 (5.6)	3 (3.5)	5 (8.6)	0.17
Neonatal hypoglycaemia	6 (4.2)	2 (2.3)	4 (6.9)	0.21
Apgar score <7 at five minutes	6 (4.2)	2 (2.3)	4 (6.9)	0.21
NICU admission	14 (9.7)	5 (5.8)	9 (15.5)	0.049*

Binary logistic regression demonstrated that high BMI was a strong predictor of several maternal and neonatal complications (Table 4). After adjustment for relevant confounders (maternal age, education, socioeconomic status, residence, and anaemia), high BMI remained significantly associated with caesarean section, HDP, GDM, induction of labour and delivery complications, including PPH, or wound infection. Among neonatal outcomes, preterm birth and NICU admission showed higher odds among high-BMI mothers.

**Table 4:** Association between maternal BMI and adverse maternal and neonatal outcomes ^*^p value < 0.05; statistically significant; ^#^odds are provided for high BMI women in relation to women with a normal BMI (reference); ^^^adjusted for maternal age, education, socioeconomic status, residence, gestational age at delivery, and anaemia. Only significant variables in the univariate logistic regression were included in the multivariate logistic regression.

Maternal and neonatal outcomes	Crude OR^# ^(95% CI); p-value	Adjusted OR^^^ (95% CI); p-value
Hypertensive disorders of pregnancy (HDP)	2.86 (1.24-6.61); 0.014^*^	2.41 (1.01-5.76); 0.02^*^
Gestational DM	4.27 (1.26-14.4); 0.003^*^	3.61 (1.03-12.6); 0.04^*^
Caesarean section	2.28 (1.71-2.53); 0.002^*^	2.23 (2.08-2.40); 0.006^*^
Induction of labour	2.76 (1.37-5.56); 0.01^*^	2.33 (1.11-4.88); 0.02^*^
Preterm delivery	2.45 (1.97-6.14); 0.03^*^	1.91 (1.47-6.49); 0.012
NICU admission	3.26 (1.22-8.68); 0.03^*^	2.88 (1.16-8.33); 0.04^*^

Overall, the results indicate that high maternal BMI is independently associated with increased risk of major maternal complications (hypertensive disorders, GDM, labour and delivery complications) as well as significant neonatal morbidity, reinforcing the importance of early risk assessment and targeted antenatal care for primigravid women with elevated BMI.

## Discussion

The present study shows that high maternal BMI is significantly associated with several adverse maternal and neonatal outcomes among primigravid women. With rising urbanisation, sedentary behaviour, and nutritional transition, India is witnessing a steady increase in overweight among women of reproductive age, making this an important clinical and public health concern.

Our study found that women with high BMI had significantly higher odds of developing HDP, which aligns with earlier findings by Shao et al. and Agarwal et al. showing a two- to four-fold increase in HDP among obese pregnant women [6,11]. Several Indian studies have reported similar associations. A study by Kutchi et al. demonstrated a significant rise in gestational hypertension among overweight and obese women [12], while a South Indian cohort from Vellore reported that maternal obesity nearly doubled the risk of preeclampsia [13]. Proposed mechanisms include endothelial dysfunction, enhanced oxidative stress, and adipokine-mediated inflammation, all of which compromise placental perfusion.

Similarly, GDM was significantly higher in the high-BMI group in our study [14,15]. Gao et al. reported a strong association between maternal overweight and GDM risk among women in Tamil Nadu [16]. Insulin resistance, reduced adiponectin, and increased leptin levels in obese individuals predispose them to dysregulated glucose metabolism, explaining this consistent association [17,18].

Labour and delivery complications were more frequent among women with high BMI in this study. These findings align with the results of Arrowsmith et al. and Shirvanifar et al. [19,20], who noted increased risks of failed induction, dysfunctional labour, and caesarean section among obese women. A study by Khalifa et al. also observed higher rates of prolonged labour and caesarean section in obese primigravid women [10]. Soft tissue dystocia, impaired myometrial contractility, and increased foetal size have been suggested as potential causes.

Neonatal outcomes, including respiratory distress, hypoglycaemia, and low Apgar scores, were more common among infants of high-BMI mothers in our study. These results align with a study by Brand et al., who found that maternal obesity is associated with compromised neonatal adaptation and metabolic instability [21]. Several Indian studies support these findings as well. A study by Gandhi et al. reported significantly higher NICU admissions among neonates born to overweight mothers, while a Hyderabad-based cohort observed increased neonatal respiratory morbidity among infants of obese mothers [22,23].

The strength of this study is its exclusive focus on primigravid women, a group at higher risk of maternal and perinatal complications. The prospective design, with complete follow-up until six weeks postpartum, enabled accurate and systematic documentation of maternal and neonatal outcomes.

The single-centre, tertiary-care setting may introduce referral bias and limit external validity. The modest sample size reduced statistical power for infrequent maternal and neonatal complications. The absence of a formal sample size calculation and the use of convenience sampling increase the potential for selection bias. Additionally, reliance on booking BMI may not fully capture true pre-pregnancy adiposity, as some women may experience early gestational weight changes before registration. Future studies should incorporate objectively recorded longitudinal weight trajectories to improve exposure assessment. Furthermore, residual confounding from unmeasured lifestyle and metabolic factors, such as dietary intake, physical activity, and baseline insulin sensitivity, cannot be excluded. These limitations could be addressed in future research through larger, multicentre studies with comprehensive data collection on behavioural, metabolic, and biochemical parameters, enabling more robust adjustment for potential confounders.

## Conclusions

This study shows that high maternal BMI measured early in pregnancy is significantly associated with several adverse outcomes among primigravid women. Clinically, routine assessment of BMI at booking and early risk stratification are essential. Women with elevated BMI should receive targeted interventions such as nutritional counselling, early glucose screening, regular blood pressure monitoring, and closer intrapartum surveillance. Strengthening preconception counselling, promoting healthy weight among young women, and integrating lifestyle and dietary support into existing reproductive and maternal health programmes are crucial. Early prevention and focused management can help reduce complications, improve pregnancy outcomes, and interrupt the intergenerational cycle of obesity.

## References

[1] (2026). Obesity and overweight. World Health Organization. https://www.who.int/news-room/fact-sheets/detail/obesity-and-overweight.

[2] (2026). International Institute for Population Sciences (IIPS) & ICF. National Family Health Survey (NFHS-5), 2019-21. Mumbai, India: IIPS.

[3] Almutairi FS, Alsaykhan AM, Almatrood AA (2024). Obesity prevalence and its impact on maternal and neonatal outcomes in pregnant women: a systematic review. Cureus.

[4] Chopra M, Kaur N, Singh KD (2020). Population estimates, consequences, and risk factors of obesity among pregnant and postpartum women in India: results from a national survey and policy recommendations. Int J Gynaecol Obstet.

[5] Ramachenderan J, Bradford J, McLean M (2008). Maternal obesity and pregnancy complications: a review. Aust N Z J Obstet Gynaecol.

[6] Shao Y, Qiu J, Huang H (2017). Pre-pregnancy BMI, gestational weight gain and risk of preeclampsia: a birth cohort study in Lanzhou, China. BMC Pregnancy Childbirth.

[7] Vinutha BR, Savithri DR (2025). Effect of pre-pregnancy body mass index on mode of delivery: a comprehensive observational study. Eur J Cardiovasc Med.

[8] Ellis JA, Brown CM, Barger B, Carlson NS (2019). Influence of maternal obesity on labor induction: a systematic review and meta-analysis. J Midwifery Womens Health.

[9] Kureshi A, Khalak R, Gifford J, Munshi U (2022). Maternal obesity-associated neonatal morbidities in the early newborn period. Front Pediatr.

[10] Khalifa E, El-Sateh A, Zeeneldin M (2021). Effect of maternal BMI on labor outcomes in primigravida pregnant women. BMC Pregnancy Childbirth.

[11] Agrawal S, Maitra N (2016). Prediction of adverse maternal outcomes in preeclampsia using a risk prediction model. J Obstet Gynaecol India.

[12] Kutchi I, Chellammal P, Akila A (2020). Maternal obesity and pregnancy outcome: in perspective of new Asian Indian guidelines. J Obstet Gynaecol India.

[13] Jacob SE, Ebenezer ED, Tirkey RS (2022). Is new onset hypertension in obese women more likely to be Gestational Hypertension? - a retrospective study. J Family Med Prim Care.

[14] Hedderson MM, Gunderson EP, Ferrara A (2010). Gestational weight gain and risk of gestational diabetes mellitus. Obstet Gynecol.

[15] Samahy MH, Elbarbary NS, Elmorsi HM (2015). Current status of diabetes management, glycemic control and complications in children and adolescents with diabetes in Egypt. Where do we stand now? And where do we go from here?. Diabetes Res Clin Pract.

[16] Gao S, Zhao D, Qi Y, Wang M, Zhao F, Sun J, Liu J (2017). The association between serum ferritin levels and the risk of new-onset type 2 diabetes mellitus: a 10-year follow-up of the Chinese Multi-Provincial Cohort Study. Diabetes Res Clin Pract.

[17] Hunt JS, Petroff MG (2013). IFPA senior award lecture: reproductive immunology in perspective - reprogramming at the maternal-fetal interface. Placenta.

[18] Angeliki A, Dimitrios P, Chara T (2020). Maternal obesity and its association with the mode of delivery and the neonatal outcome in induced labour: implications for midwifery practice. Eur J Midwifery.

[19] Arrowsmith S, Wray S, Quenby S (2011). Maternal obesity and labour complications following induction of labour in prolonged pregnancy. BJOG.

[20] Shirvanifar M, Ahlqvist VH, Lundberg M (2024). Adverse pregnancy outcomes attributable to overweight and obesity across maternal birth regions: a Swedish population-based cohort study. Lancet Public Health.

[21] Brand JS, Czene K, Eriksson L, Trinh T, Bhoo-Pathy N, Hall P, Celebioglu F (2013). Influence of lifestyle factors on mammographic density in postmenopausal women. PLoS One.

[22] Gandhi S, Chauhan K, Rajan P, Wanjari M, Sangoi L, Sangoi R (2024). Impact of maternal body mass index on pregnancy outcomes among Indian women. Bioinformation.

[23] Koripadu S, Pusapati S, Kakarala G (2022). Maternal factors influencing very low birth weight babies: a hospital-based case-control study from India. Casp J Pediatr.

